# Calretinin: from a “simple” Ca^2+^ buffer to a multifunctional protein implicated in many biological processes

**DOI:** 10.3389/fnana.2014.00003

**Published:** 2014-02-05

**Authors:** Beat Schwaller

**Affiliations:** Anatomy, Department of Medicine, University of FribourgFribourg, Switzerland

**Keywords:** calretinin, calcium signaling, calcium sensor, calcium buffer, neuron excitability

## Abstract

The hexa-EF-hand Ca^2+^-binding protein calretinin (CR) is predominantly expressed in specific neurons of the central and peripheral nervous system. However, CR expression is also observed in non-neuronal cells, e.g., during embryonic development and in mesothelioma cells. Of the 6 EF-hand domains, 5 are functional; the first 4 domains form 2 pairs showing high cooperativity within a pair that results in non-linear modulation of intracellular Ca^2+^ signals by CR. EF-hand domain 5 has a low affinity and represents the identified interaction site with CR-binding partners present in mouse cerebellar granule cells. CR binding to other targets including the pore-forming α_1_ subunit of the Ca^2+^ channel Ca_*V*_2.1, as well as to huntingtin indicates additional Ca^2+^ sensor functions besides the well-known Ca^2+^-buffering functions. The absence of CR in cerebellar granule cells of CR^−/−^ mice results in increased excitability and altered firing of Purkinje cells and promotes cerebellar 160-Hz oscillations impairing motor coordination. The putative role of CR in neuroprotection is still highly discussed. Altogether, CR emerges as a multi-functional protein also associated with development, i.e., cell proliferation, differentiation, and cell death.

## Basic facts about calretinin (CR)

Calretinin (CR; human gene symbol: *CALB2*), calbindin D-28k (CB; *CALB1*) and secretagogin (SCGN; *SCGN*) represent the 3 members of the hexa-EF-hand protein family, also named the calbindin sub-family [see recent reviews on CR (Camp et al., 2009), CB (Schmidt, 2012) and SCGN (Alpar et al., 2012)]. They all contain 6 structural motifs named EF-hand Ca^2+^-binding domains. Each domain consists of an alpha-helix of approximately 10 amino acids, a Ca^2+^-chelating loop of 12 amino acids and a second alpha-helix that is oriented perpendicular to the first one (for more details on the EF-hand structure, see (Schwaller, 2010). CR (M_r_ 31 kDa) initially discovered in the retina, thus the name: calcium + retina = CR consists of 271 amino acids in many species and is highly conserved; the number of amino acids varies from 269 (e.g., *Gallus gallus*; Chicken) to 273 (e.g., *Monodelphis domestica*; Gray short-tailed opossum). CR is also expressed in zebrafish (*Danio rerio)* and an invertebrate ortholog named calbindin 53E (*Cbp53E*; previously *calbindin-32*) exists in *Drosophila melanogaster* that shares the highest sequence identity with CR (Reifegerste et al., 1993). In CR, the first 5 EF-hand domains are capable of binding Ca^2+^ ions, while the sixth one is inactive (Stevens and Rogers, 1997; Schwaller et al., 1997). Moreover the Ca^2+^-binding affinity for site 5 is very low (K_D_: 36 μM) (Faas et al., 2007) indicating that this site is expected to be rarely in the Ca^2+^-bound form in the cytoplasmic compartment except in microdomains close to Ca^2+^ channels. The other 4 functional Ca^2+^-binding sites form 2 similar pairs likely consisting of domains 1 and 2, as well as 3 and 4 showing strong cooperativity within a pair (Faas et al., 2007). The apparent K_D_ (K_D, app_) for the 4 sites is 1.4–1.5 μM with high cooperativity (n_H_ of 1.9; for more details on CR's properties, see Table 1). This property results in non-linear Ca^2+^ regulation in cells due to the presence of CR. In a situation when the intracellular Ca^2+^ concentration [Ca^2+^]_i_ is at resting (basal) levels of 50–100 nM, then upon a brief and limited increase in [Ca^2+^]_i_, CR behaves like a typical slow-onset buffer (EGTA). However, if the same increase occurs at elevated [Ca^2+^]_i_, in the order of 1 μM, when the first site of a pair is in the Ca^2+^-bound form, cooperativity sets in and CR functions almost like the fast buffer BAPTA (for more details on this behavior, e.g., on the spatiotemporal patterns of IP_3_-evoked Ca^2+^ signals, see Dargan et al. (2004) or on CR's role modeled for a train of intracellular Ca^2+^ signals, see Figure 3 in (Schwaller, 2009). Thus, the Ca^2+^-binding kinetics of CR strongly depends on [Ca^2+^]_i_ levels at the time when another increase in [Ca^2+^]_*i*_ occurs. Besides these novel properties of Ca^2+^ binding in a protein, first described for CR, several studies in the 90's reported CR to undergo considerable Ca^2+^-dependent conformational changes, which indicated that CR might also have “Ca^2+^ sensor” functions like the prototypical sensor calmodulin (CaM). Results in support of CR acting as a Ca^2+^ sensor are presented in Section III.

**Table 1:** **Properties of calretinin (modified from Schwaller, 2009, 2010, 2012)**.

**General parameters**	**Value (range)**	**References/comments**
Amino acids	269–273; 271 in most mammals; 310^a^	Reifegerste et al., 1993; Zimmermann and Schwaller, 2002
Molecular mass (M_*r*_)	30–31 kDa	Rogers, 1987
EF-hand domains	6	Rogers, 1987
Functional Ca^2+^-specific sites	5	Stevens and Rogers, 1997; Schwaller et al., 1997
Identified CR binding partners	α_1_ subunit of Ca_V_2.1, huntingtin	Christel et al., 2012; Dong et al., 2012
**METAL BINDING PROPERTIES AND MOBILITY**		
K_D, Ca_	K_D(T)_ 28 μM	CR has 2 cooperative pairs (sites EF1-4) with indistinguishable binding properties. In the absence of Ca^2+^, the first site within a pair is in the tensed (T) state and changes upon Ca^2+^ binding of the first site within a pair to the relaxed (R) state. These results were obtained by flash photolysis experiments Faas et al., 2007
	K_D(R)_ 68 nM	
	K_D(app)_ 1.4 μM	
	EF5: 36 μM^b^	
K_D, Mg_	4.5 mM	Stevens and Rogers, 1997
k_on, Ca_ (μM^−1^s^−1^)	Tensed (T) sites: 1.8	Faas et al., 2007
	Relaxed (R) sites: 310	
	EF-hand domain 5: 7.3	
Cooperativity	Yes n_H_ ≈ 1.3–1.9	Stevens and Rogers, 1997; Faas et al., 2007
Mobility D_Cabuffer_ (μm^2^s^−1^) in H_2_O	120 ± 1 (means ± s.e.m.)	Arendt et al., 2013
D_10 ms_ (μm^2^s^−1^)^c^	3.2 (IQR 1.6–5.9)	
**INTRACELLULAR CONCENTRATION IN**		
Frog saccular hair cells	1.2 mM	Edmonds et al., 2000
Rat outer hair cells	35 μM	Hackney et al., 2005
Rat inner hair cells	20 μM	Hackney et al., 2005
Mouse cerebellar granule cells	30–40 μM^d^; 0.7–1.2 mM^e^	Gall et al., 2003

a Drosophila melanogaster calbindin 53E (previously calbindin-32) shows the highest sequence homology to calretinins of different species.

b A lower affinity for EF5 (K_D_: 0.5 mM) was determined with the flow dialysis method (Schwaller et al., 1997).

c Diffusion determined in cerebellar granule cell dendrites by an anomalous subdiffusion model.

d Based on BAPTA concentration (150 μM) needed to restore granule cell excitability in CR^−/−^ cells.

e Based on numerical simulations of buffered Ca^2^+ diffusion near a single Ca^2^+ channel or a large cluster of Ca^2^+ channels (Saftenku, 2012).

Up to date, no structural data of full-length CR have been reported. However, the NMR structure of the N-terminal 100 amino acids of rat CR (Palczewska et al., 2001) embracing EF-hand domains 1 and 2 are very similar to the NMR solution structure of the corresponding domains in rat CB (Kojetin et al., 2006). Together with the similar results from limited proteolysis experiments obtained with CR and CB, this suggests that the overall structure of hexa-EF-hand proteins might be rather similar.

## Regulation of calretinin expression

**Figure 1:**
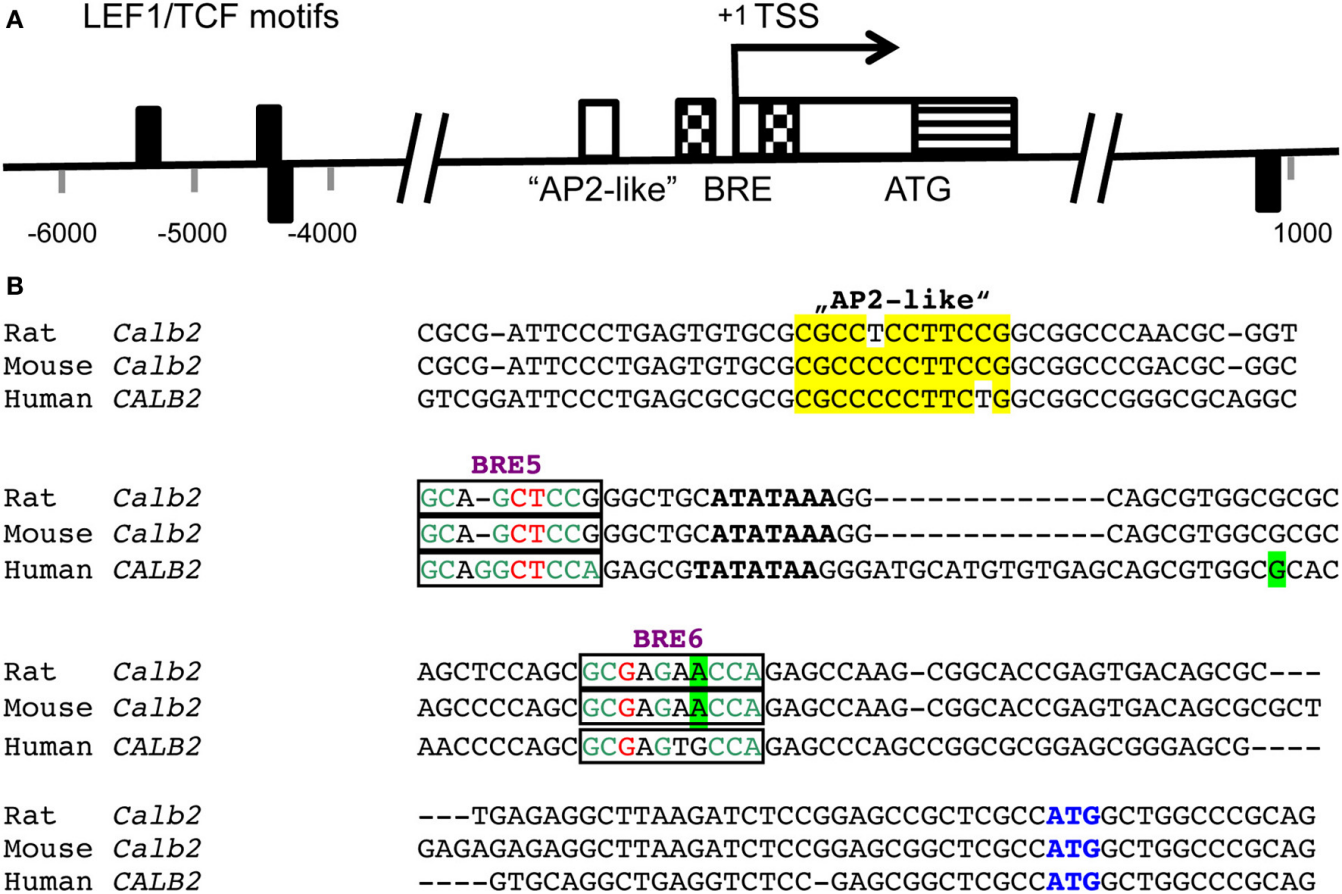
**(A)** Positions of LEF1/TCF motifs (black rectangles) are present within conserved (human/rat) non-coding regions flanking the transcription start site (TSS; pos. +1) of the CALB2/Calb2 genes and are depicted either above or below the axis depending on the strand (modified from Wisniewska et al., 2012). The consensus sequence recognized by the DNA-binding domain of LEF1/TCFs is WWCAAAG (W is either A or T). Nucleotide numbering is shown with respect to the TSS. In the region −90/−80 of the mouse Calb2 gene, an “AP2-like” element (white box) is responsible for neuron-specific expression of the transcript (Billing-Marczak et al., 2002). A bipartite butyrate-responsive element (BRE; checkered boxes) surrounds the TATA box and the TSS (Haner et al., 2010). The non-coding part of exon 1 is shown as a white box and the coding region including the ATG start codon as a striped box. **(B)** Sequence comparison of the rat, mouse and human Calb2 gene around the TSS. The “AP2-like” region is boxed in yellow. The TATA box is marked in bold and the BREs 5 and 6 acting as butyrate-responsive repressors in colon cancer cells are boxed. The most highly conserved nucleotides in the BREs (consensus sequence: GCGGGCTCCA) are shown in green, the less conserved ones in red and the nucleotides not conforming to the consensus sequence are shown in black. The TSS (+1) is boxed in green. The start codon ATG is marked in blue.

Still relatively little is known on the mechanisms of regulation of CR expression in various tissues; altered CR expression levels have been reported as the consequence of experimental manipulations or are associated with certain diseases in humans and/or animal models of these diseases [for more details, see Schwaller, 2009, 2010, 2012]. Based on the substantial sequence homology in the promoter region including the TATA and CAAT boxes of the human *CALB2* and mouse *Calb2* gene (Strauss et al., 1997), it is reasonable to assume that CR expression is regulated in a similar manner in the two species, although species differences in CR expression have been reported before. Neuron-specific “CR-like” expression of a luciferase reporter gene in cortical cultures is achieved in the presence of the mouse *Calb2* promoter region from −115/+54. The 5′ region of this promoter fragment (−115/−71) selectively binds a nuclear protein present in cerebellar granule cells and contains an “AP2-like” element (−90/−80 bp; Figure 1). This element is essential for the neuron-specific reporter expression (Billing-Marczak et al., 2002). The same “AP2-like” element doesn't affect transcriptional activity in human colon carcinoma and mesothelioma cells indicating that CR expression in neurons and non-neuronal cell types is differently regulated (Billing-Marczak et al., 2004). In human colon cancer cells, CR expression is downregulated by butyrate (Marilley et al., 2001), a substance derived from intestinal fermentation of dietary fibers by bacteria. Butyrate, a known modulator of histone acetylation, inhibits the cell cycle and leads to enterocyte differentiation. Of the several putative butyrate-responsive elements (BREs) present in the human *CALB2* promoter, two elements embracing the TATA box act as butyrate-sensitive repressor elements in colon cancer cells, but not in cells of mesothelial origin (Figure 1; Haner et al., 2010). This supports the notion of cell type-specific *CALB2* regulation. The rat *Calb2* promoter region contains 3 binding motifs for the transcription factor LEF1/TCF that binds to β-catenin via its N-terminal region (Figure 1); β-catenin, not directly binding to DNA, contains a strong transactivation domain and is highly expressed in thalamic neurons. Down-regulation of β-catenin by its negative regulator Axin2 significantly reduces CR expression in cultured rat thalamic neurons indicating that β-catenin is a positive regulator of the *Calb2* gene (Wisniewska et al., 2012). In addition, several transcripts exist from the human *CALB2* gene (Schwaller et al., 1995), which are present in several colon cancer cell lines (Gander et al., 1996) and in tumor tissue from primary colon tumors (Schwaller et al., 1998). One splice variant with deletion of exons 8 and 9 results in a truncated protein named CR-22k of 192 amino acids, which is expressed in certain tumors (Schwaller et al., 1998); the other transcript (deletion of exon 8) is currently known to exist only at the level of mRNA (Schwaller et al., 1995) and might have a function as an RNA molecule, possibly as a target for micro (mi)RNA or acting as a long non-coding (lnc) RNA.

## The identification of calretinin-interacting targets supports CR's role as a Ca^2+^ sensor

The finding that purified CR undergoes significant Ca^2+^-dependent conformational changes *in vitro* (Kuznicki et al., 1995; Schwaller et al., 1997), together with the observation that CR immunoreactivity in chick brainstem auditory neurons changes from diffuse cytosolic staining to intense localized staining beneath the plasma membrane, which occurs together with the onset of spontaneous activity (Hack et al., 2000), suggested already in the late 90's that CR might have additional Ca^2+^-sensor functions (Billing-Marczak and Kuznicki, 1995). Furthermore, CR was shown to be present in membrane fractions of rat cerebellum (Winsky and Kuznicki, 1995) and to bind to cytoskeletal elements in WiDr colon cancer cells (Marilley and Schwaller, 2000). In support of the presence of CR targets, CR translocates from the cytosol to the nucleus in a vitamin D_3_-dependant and/or butyrate-dependent way in colon cancer cells *in vitro*, also indicating that CR might have nuclear interaction partners (Schwaller and Herrmann, 1997). Recently, in two studies CR targets were identified (Christel et al., 2012; Dong et al., 2012) and moreover, the interacting domain of CR that leads to a decreased Ca^2+^-dependent mobility of CR in cerebellar granule cells was identified (Arendt et al., 2013). A yeast-two-hybrid screen with CR as bait, identified a consensus, strongly basic peptide sequence H(R/K)HRRR(E/D) as a putative CR-binding (CRB) motif (Christel et al., 2012). This motif is present in multiple copies (CRB1-5) in the cytoplasmic linker region between domains II and III of the channel-forming alpha 1 subunit of the high-voltage activated Ca^2+^ channel Ca_*V*_2.1 (P/Q type). This channel is regulated in a dual fashion by Ca^2+^ ions, showing both, Ca^2+^-dependent inactivation (CDI) and Ca^2+^-dependent facilitation (CDF); both mechanisms influence synaptic plasticity in the nervous system. In cells expressing Ca_*V*_2.1 *in vitro*, co-expression of CR inhibits CDI and enhances CDF via a direct interaction with the α_1_2.1 subunit. The channel subunit α_1_2.1 co-immunoprecipitates with CR antibodies using either extracts from HEK293T cells transfected with CR and Ca_*V*_2.1 or mouse cerebellar extracts. In mouse cerebellum, both CR and Ca_*V*_2.1 are strongly expressed in granule cells and the absence of CR in CR^−/−^ mice causes impairment in motor control (Schiffmann et al., 1999). This impairment is essentially caused by CR's absence in granule cells, since the motor phenotype can be rescued by selective re-expression of CR in granule cells (Bearzatto et al., 2006). Thus, the direct modulation of Ca_*V*_2.1 by CR affects intracellular Ca^2+^ signaling and probably also neuronal excitability via a mechanism that is different from CR's previously proven Ca^2+^ buffering function as discussed in Section IV. The interaction between CR and interacting partners likely including Ca_*V*_2.1 was studied in granule cells by fluorescence recovery after photobleaching (FRAP). The diffusion of fluorescence-labeled CR molecules is much slower than the one of freely diffusible molecules (fluorescein dextrans) of comparable size (Arendt et al., 2013). Moreover, during a burst of action potentials (APs) that leads to an increase in dendritic [Ca^2+^]_*i*_, CR's mobility is further decreased, indicative of Ca^2+^-dependent interactions. Addition of a peptide consisting of EF-hand 5 of CR to granule cells, considerably increases CR's mobility implicating that the CR interactions occur mainly via the region of EF-hand 5, the Ca^2+^-binding site with very low affinity. Estimations on the density (concentration) of Ca_*V*_2.1 channels and CR in granule cells indicate that channel numbers are too low to account for the strong effect on CR's mobility implicating additional, yet unidentified CR-binding partners in these neurons. A binding partner interacting with CR was found to be huntingtin (Htt), identified by tandem affinity purification (Dong et al., 2012). Binding to CR is even stronger with a mutant form of Htt characterized by a polyglutamine (polyQ) region that is typical for Huntington's disease (HD). In neuronal cultures, CR colocalizes with Htt and a CR/Htt complex can be isolated by co-immunoprecipitation. In CR-overexpressing HEK293 cells, levels of phosphorylated AKT (p-AKT) are increased. At the functional level, CR overexpression reduces mHtt-related H_2_O_2_ cytotoxicity in various HD *in vitro* models. This might be directly linked to CR's capacity to decrease [Ca^2+^]_*i*_ in these cells and/or to indirectly increase levels of p-AKT considered as a pro-survival signal. On the other hand, CR down-regulation by shRNA enhances mHtt-mediated neuronal cell death. Based on their findings, the authors conclude that “CR may be a potential therapeutic target for treatment of HD.” A link between CR and p-AKT was reported before; increased expression levels of CR strongly correlate with increased resistance to asbestos cytotoxicity in immortalized Met-5A mesothelial cells (Henzi et al., 2009). This protective effect is abrogated in the presence of phosphatidylinositol 3-kinase (PI3K) inhibitors, in support of the above findings that increased PI3K/AKT signaling (increased p-AKT) caused by CR up-regulation favors cell survival. Thus, in the case of CR-expressing mesothelial and mesothelioma cells, CR or more precisely its down-regulation, might be viewed as a potential new target/strategy for malignant mesothelioma therapy (Blum and Schwaller, 2013).

## The effect of calretinin on intracellular Ca^2+^ signaling

The particular Ca^2+^-binding properties of CR together with its mobility differently affect intracellular Ca^2+^ signals, however only to a measurable extent, if present at a sufficiently high concentration, typically in the range of tens of μM in neurons. Generally, lower CR concentrations (≈1 μM) don't affect Ca^2+^ signals and e.g., don't protect PC12 cells against Ca^2+^ overload induced by ionophore treatment or trophic factor deprivation (Kuznicki et al., 1996). Effects of CR on Ca^2+^ signals are often deduced from comparing signals in neurons from WT and CR^−/−^ mice (Schmidt et al., 2013) or when overexpressing or down-regulating CR in cell culture models (Billing-Marczak et al., 1999; D'Orlando et al., 2001; Pecze et al., 2013). CR's particular properties, i.e., its low Ca^2+^ occupancy at resting [Ca^2+^]_*i*_ together with the high co-operativity resulting in non-linear binding properties in a setting in which neurotransmitter release depends supralinearly on Ca^2+^ (e.g., in parallel-fiber (PF) terminals onto Purkinje cells) result in considerable nanodomain Ca^2+^-buffering by CR. As a consequence, a minor increase in the amplitude of AP-evoked Ca^2+^ signals in CR^−/−^ PF boutons results in a considerably higher release probability (Schmidt et al., 2013). CR-deficient cerebellar granule cells are characterized by faster APs and, when electrically stimulated to generate repetitive spike discharges, show enhanced frequency increase with injected currents, i.e., increased excitability (Gall et al., 2003). The excitability can be reverted to the situation seen in WT cells, by loading the cells with the fast buffer BAPTA (150 μM) strongly indicating that the “fast” Ca^2+^ buffering function of CR is most likely responsible for limiting granule cell excitability. From these experiments it was also deduced that the CR concentration in these neurons is in the order of 40 μM, based on CR's 4 high-affinity Ca^2+^-binding sites; this estimation is in line with modeling studies on CR function (Roussel et al., 2006). However, other models taking into account CR's cooperativity of Ca^2+^ binding resulting in a delayed equilibration with Ca^2+^ predict the concentration of CR to be even higher, in the order of 0.7–1.2 mM (Saftenku, 2012), a value estimated to be present in frog saccular hair cells (Edmonds et al., 2000). However, in this model the modulation of the main voltage-activated Ca^2+^ channel in granule cells, Ca_*V*_2.1, by CR (Christel et al., 2012) was not taken into account. Thus, the precise concentration of CR in granule cells has to be determined yet, possibly by a *in situ* calibration method as previously used for the determination of the concentration of CB (≈40 μM) in hippocampal granule cells (Muller et al., 2005) or of PV in DG basket cells (11.9 ± 1.6 μ M) or in cerebellar basket cells (563 ± 66 μ M) (Eggermann and Jonas, 2012). The particular biophysical properties of CR also acting as a slow-onset Ca^2+^ buffer are best appreciated from studies in *Xenopus* oocytes (Dargan et al., 2004). Photo-release of inositol 1,4,5 triphosphate (IP_3_) evokes Ca^2+^ signals that are differently modulated by endogenous or synthetic Ca^2+^ buffers (Dargan and Parker, 2003). In the presence of slow buffers such as PV or EGTA, global Ca^2+^ signals are fragmented into local Ca^2+^ puffs, resulting from Ca^2+^ release from discrete clusters of IP_3_ receptors, while low concentrations of fast buffers (CB, BAPTA) decrease the amplitude of Ca^2+^ signals and favor “globalization” of spatially uniform Ca^2+^ signals, in particular, at high [IP_3_]. Interestingly, puffs are observed in the presence of CR at low stimulation intensities, i.e., at low [IP_3_], an effect never occurring in the presence of CB or BAPTA. Thus, under conditions of small elevations in [Ca^2+^]_*i*_ from resting Ca^2+^ levels, CR has properties of a slow Ca^2+^ buffer such as PV or EGTA.

## Calretinin expression is linked to neuronal development

The detailed analyses of temporal and spatial expression of CR in the brain is the major focus of this Frontiers series, has been summarized in several papers and reviews Arai et al. (1991); Jacobowitz and Winsky (1991); Hof et al. (1999); Barinka and Druga (2010) and is thus not covered in this mini-review. Recent findings on CR expression (often transient) and neurogenesis are briefly summarized. Olfactory receptor neurons are generated throughout lifetime and are characterized by a short period of CR expression just before these neurons are fully mature (Wei et al., 2013), yet the functional significance is currently unknown. Also mouse adult hippocampal neurogenesis, more precisely, the early postmitotic stage of dentate gyrus (DG) granule cell development is characterized by transient CR expression (Brandt et al., 2003). This stage coincides with onset of differentiation and absence of CR in the immature early postmitotic granule cells of CR^−/−^ mice (systematic name: Calb2^tm1Map^) “causes an early loss in proliferative capacity of the subgranular zone that is maintained into adult age, when it has a further impact on the migration/survival of newborn granule cells” (Todkar et al., 2012). Interestingly, when in WT mice newborn cells are functionally integrated in the DG granule cell network, CR expression stops and is changed to CB, the typical marker for adult DG granule cells. The functional consequences of this swap from CR to CB expression for granule cell physiology are currently unknown, as well as the mechanisms that lead to such a change. Nonetheless, it indicates Ca^2+^-binding protein-specific functions that cannot be shared and/or substituted even by apparently very similar proteins such as CB and CR.

## What is the physiological role of calretinin?

As was previously reported for CB (Schmidt, 2012), CR certainly has more than one function, depending on various parameters including cell type (neurons vs. non-excitable cells including tumor cells), stages of development (adult vs. developmental stages) and probably also different neuronal subtypes. For some proposed roles of CR, e.g., a role in neuroprotection, the proportion of reports, mostly obtained in correlative studies, in favor or against such a role is almost 50:50 [for more details, see (Schwaller, 2009, 2010)], clearly necessitating more studies directly addressing this putative function of CR. Here, just the most important results obtained in CR^−/−^ mice are summarized. The decreased LTP in the DG is thought to be the result of an increased excitatory drive from CR-depleted mossy cells onto hilar interneurons (Schurmans et al., 1997). Most findings on the function of CR are derived from studies in the cerebellum, where CR is expressed in cerebellar granule cells. Their increased excitability in the absence of CR (Gall et al., 2003) is linked to the altered firing properties (Cheron et al., 2000) and likely Ca^2+^ homeostasis of Purkinje cells and the emergence of cerebellar 160-Hz oscillations (Cheron et al., 2004) that result in impairment in motor coordination (Schiffmann et al., 1999), for more details, see Schwaller (2009). Thus, CR expression in granule cells appears necessary for correct computation that is crucial for the coding and storage of information in the cerebellum. Of note, there is currently no data available on CR's function in cortical interneurons, e.g., derived from CR^−/−^ mice. I am also not aware that anybody has attempted to manipulate CR expression levels of cortical interneurons, e.g., by shRNA and to investigate the functional consequences of CR down-regulation.

In CR-expressing mesothelial cells, CR down-regulation causes a G_1_ block and in mesothelioma-derived cells leads to apoptosis via strong activation of the intrinsic caspase 9-dependent pathway (Blum and Schwaller, 2013). A rather similar effect is also seen in CR-expressing colon cancer cells (Gander et al., 1996) indicating a role for CR in cell cycle regulation, proliferation, possibly differentiation and cell death. These findings are in line with transient CR expression during development, whether in the nervous system or in other tissues including mesenchymal tissue (Gangji et al., 1994). In summary, we have just started to unravel the likely many functions of CR in different settings and there are still plenty of interesting aspects on CR's function(s) to be discovered.

### Conflict of interest statement

The author declares that the research was conducted in the absence of any commercial or financial relationships that could be construed as a potential conflict of interest.
